# Genetic dissection of grain zinc concentration in spring wheat for mainstreaming biofortification in CIMMYT wheat breeding

**DOI:** 10.1038/s41598-018-31951-z

**Published:** 2018-09-10

**Authors:** Govindan Velu, Ravi Prakash Singh, Leonardo Crespo-Herrera, Philomin Juliana, Susanne Dreisigacker, Ravi Valluru, James Stangoulis, Virinder Singh Sohu, Gurvinder Singh Mavi, Vinod Kumar Mishra, Arun Balasubramaniam, Ravish Chatrath, Vikas Gupta, Gyanendra Pratap Singh, Arun Kumar Joshi

**Affiliations:** 1 International Maize and Wheat Improvement Center (CIMMYT), Mexico, D.F. Apdo Postal 6-641, Mexico; 2 Cornell University, Ithaca, NY 14850 USA; 3 Flinders University, School of Biological Sciences, Adelaide, 5001 Australia; 4 Punjab Agricultural University (PAU), Ludhiana, India; 5 Banaras Hindu University (BHU), Varanasi, India; 6 Indian Institute of Wheat and Barley Research (IIWBR), Karnal, India; 7 CIMMYT, Global Wheat Program, New Delhi, India

## Abstract

Wheat is an important staple that acts as a primary source of dietary energy, protein, and essential micronutrients such as iron (Fe) and zinc (Zn) for the world’s population. Approximately two billion people suffer from micronutrient deficiency, thus breeders have crossed high Zn progenitors such as synthetic hexaploid wheat,* T. dicoccum, T. spelta*, and landraces to generate wheat varieties with competitive yield and enhanced grain Zn that are being adopted by farmers in South Asia. Here we report a genome-wide association study (GWAS) using the wheat Illumina iSelect 90 K Infinitum SNP array to characterize grain Zn concentrations in 330 bread wheat lines. Grain Zn phenotype of this HarvestPlus Association Mapping (HPAM) panel was evaluated across a range of environments in India and Mexico. GWAS analysis revealed 39 marker-trait associations for grain Zn. Two larger effect QTL regions were found on chromosomes 2 and 7. Candidate genes (among them zinc finger motif of transcription-factors and metal-ion binding genes) were associated with the QTL. The linked markers and associated candidate genes identified in this study are being validated in new biparental mapping populations for marker-assisted breeding.

## Introduction

More than two billion people worldwide do not get enough essential vitamins and minerals in their diets^1^. Inadequate Zn intake affects 17.3% of the world’s population, mostly across Asia and Africa^2^, and is responsible for the deaths of 433,000 children under the age of five each year^3^. The impact of micronutrient deficiencies is most serious in women of reproductive age (especially pregnant women) and children under the age of five, due to their increased micronutrient requirements^4,5^.

Wheat is an important staple food crop that provides about 20% of daily dietary energy and protein and is a major source of essential micronutrients such as Fe and Zn^6^. For more than 70 years, the International Maize and Wheat Improvement Center (CIMMYT) has bred wheat for improved grain yield potential and processing quality, resulting billions of dollars of benefits to farmers and consumers over this period^7^. The development and dissemination of high-yielding, nutrient-rich wheat varieties is a cost-effective and sustainable solution for minimizing nutrition gaps in remote rural households^8^. However, breeding for enhanced Zn concentrations is quite challenging due to the complexity of genetic and metabolic networks controlling the Zn homeostasis^9–11^. Moreover, differences in wheat plants Zn use efficiency, variability of translocation into the grain, and genotype-dependent source-sink relations can affect internal mobilization patterns of these micronutrients^12^.

Nutritional quality traits (such as Zn and Fe) tend to be multigenic. Breeding strategies are therefore focusing on novel approaches to broaden the genetic base using wild relatives and landraces, and identifying genetic control and their effects^13^. The biofortification breeding program at CIMMYT emphasizes the development of new spring bread wheat germplasm that combines wide adaptability, high yields, high grain Zn concentration, better processing quality, disease resistance, and stress tolerance. Targeted breeding for improved nutritional quality has successfully incorporated 30–40% more grain Zn into CIMMYT-derived high yielding wheat genotypes^14,15^. Yet, dissecting the genetic bases and exploring important chromosomal loci of nutritional quality traits is extremely important for further improving wheat nutritional quality.

Genome-wide association studies (GWAS) are one of the main approaches for dissecting the genetic bases of complex traits^16–18^. To date, numerous QTL studies have attempted to uncover the genetic basis of grain Zn and Fe concentrations in wheat^19–23^. However, the traditional QTL mapping approach is limited to the bi-parental population used and identifies QTL positions at low resolution. In wheat, GWAS have been widely used to analyze the genetic control of complex traits. Advantages of GWAS over traditional QTL mapping include increased QTL resolution, allele coverage, and the potential to use large sets of natural germplasm resources such as landraces, elite cultivars, and advanced breeding lines. In wheat, few studies have used GWAS to dissect the genetics of end-use quality traits^24^. To our knowledge, this is the first study that applies GWAS to dissect the genetics of grain Zn concentration in CIMMYT germplasm.

Our study used the Illumina iSelect 90 K Infinitum SNP array^25^ to genotype and analyze a diverse panel of 330 high zinc wheat lines for grain micronutrients across multiple environments. The study aimed to identify molecular markers associated with grain Zn concentrations and to identify associated candidate genes using recently-published wheat reference sequence information. The results will facilitate marker-assisted breeding for enhanced grain Zn in wheat.

## Results

**Figure 1:**
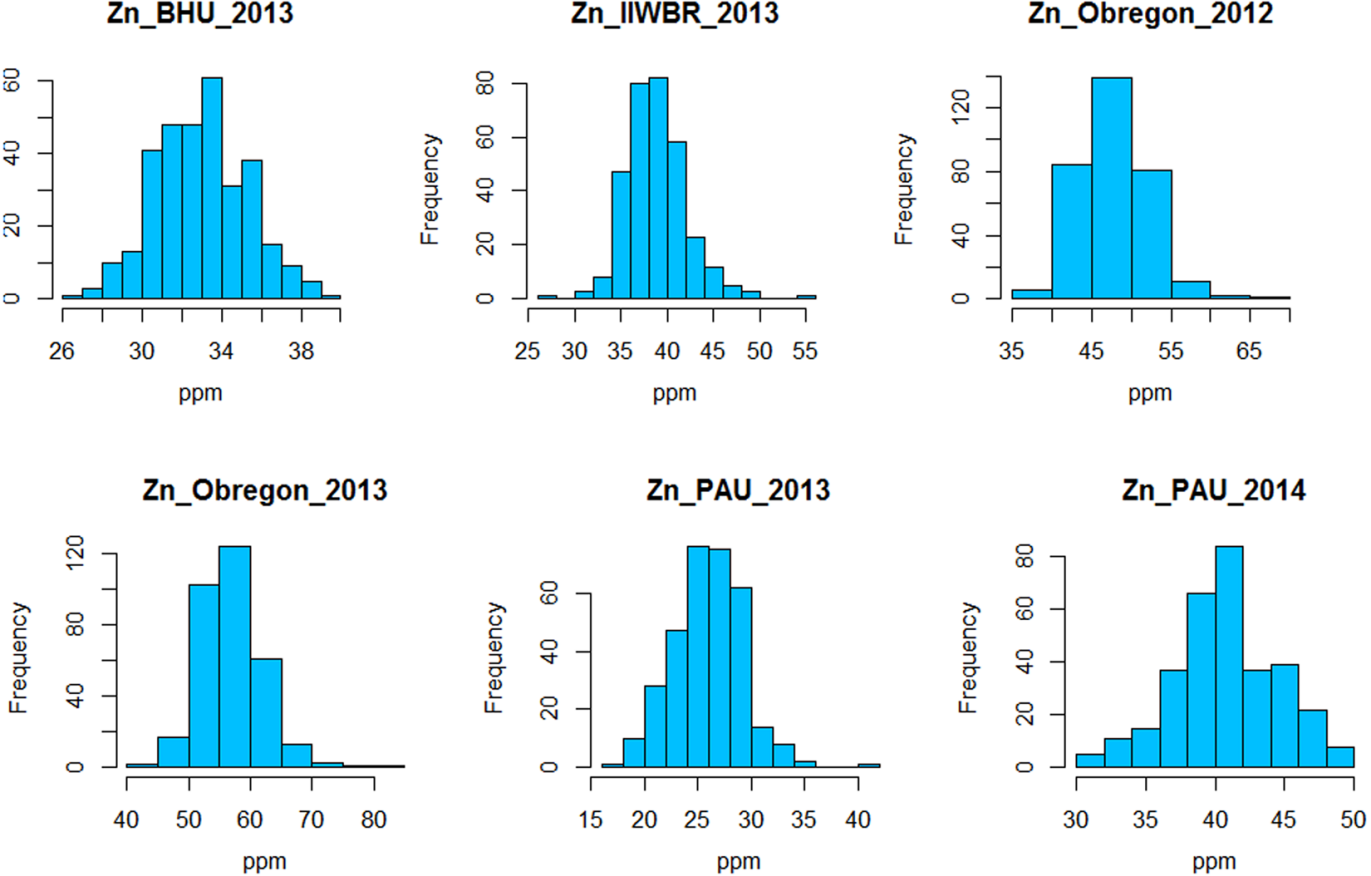
Frequency distribution of grain  Zn concentration across six different environments.

The combined analysis of variance across six environments showed significant differences between genotypes and significant genotype × environment interaction effects for grain Zn concentration, which was evident from the moderate level of broad-sense heritability (*H*^2^ = 0.6) for grain Zn. Continuous variation of grain Zn concentration (the primary target trait) was observed in all environments (Fig. 1). Across both seasons in Ciudad Obregon, Mexico, there was much wider variation in grain Zn concentration at CENEB (high Zn environment), compared to intermediate grain Zn levels at IIWBR and lower levels at PAU and BHU (low Zn environments) was observed. Differences in grain Zn between environments were probably caused by different soil Zn levels at each test location.

Large variation for grain Zn concentration was observed in crop seasons Y-2012 and Y-2013, with a range of 35.5–67.7 mg/kg and 42.5–80.3 mg/kg, respectively, and mean of 47.8 mg/kg and 56.9 mg/kg, respectively. Moderate variation was observed at IIWBR (27.2–54.6 mg/kg, mean 38.8 mg/kg), and low variation was observed in low Zn environments at BHU (26.7 to 39.4 mg/kg) and PAU-2013 (17.8 to 40.5 mg/kg) and PAU 2014 (27.9 to 50.9 mg/kg) (Fig. 1).

### GWAS for grain zinc and agronomic traits

**Figure 2:**
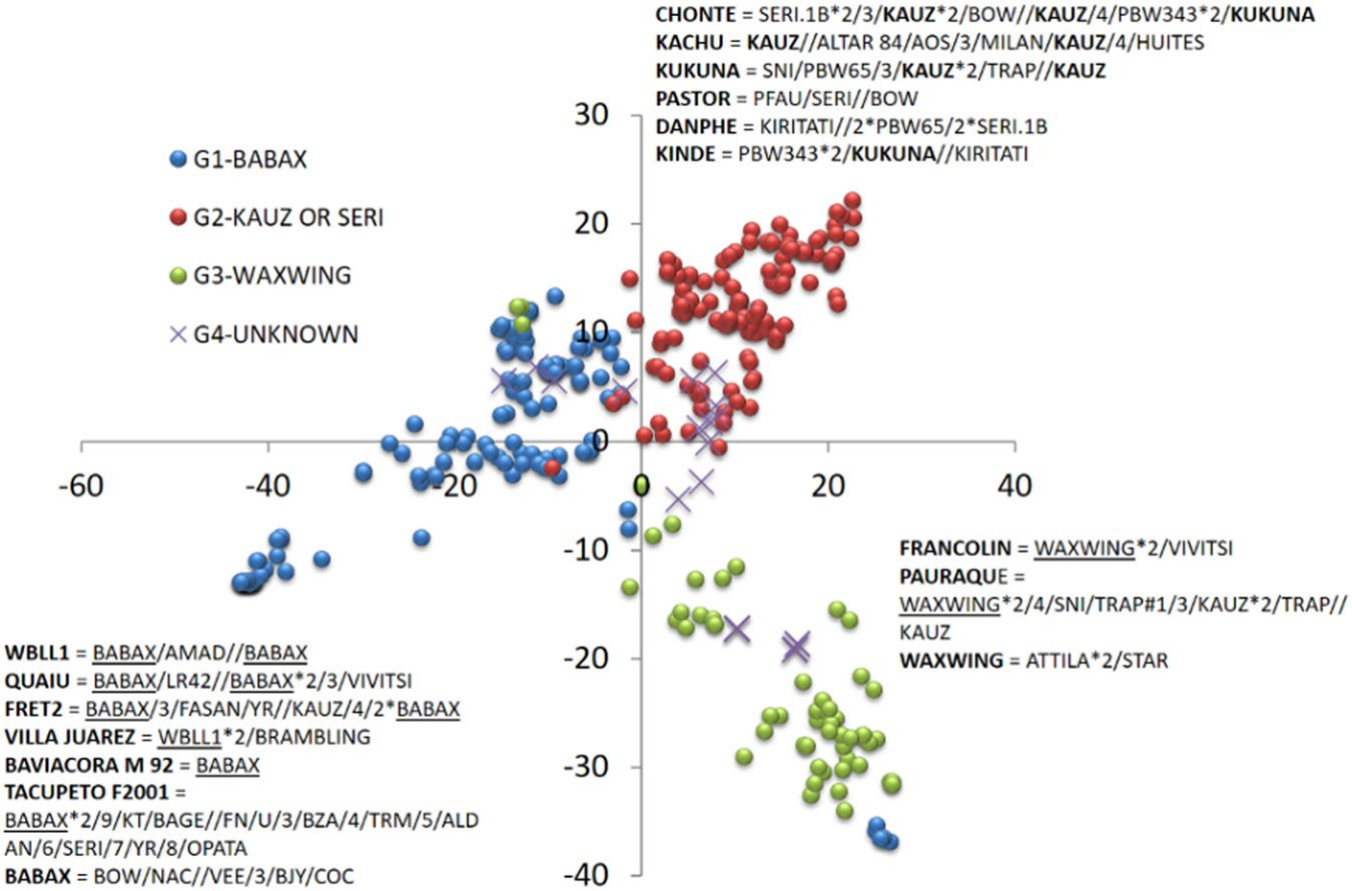
Principal component analysis of the HPAM lines. Examples of pedigrees for a few lines in each group are given.

Principal component analysis indicated four groups in the HPAM panel (Fig. 2). The first principal component separated a group of Veery (Babax)-derived lines (G1) from a second large group of lines with the parents Kauz or Seri in their pedigree (G2). Lines with Attila and Waxwing in their genetic background formed a third group (G3). The fourth group (G4) was formed by lines unrelated to any of the previous three groups. Since the lines included in the panel were selected based on pedigree information, the partitioning of the genetic diversity in subgroups remained a distinct feature of the panel, similar to that observed in other elite wheat collections^26^.

**Figure 3:**
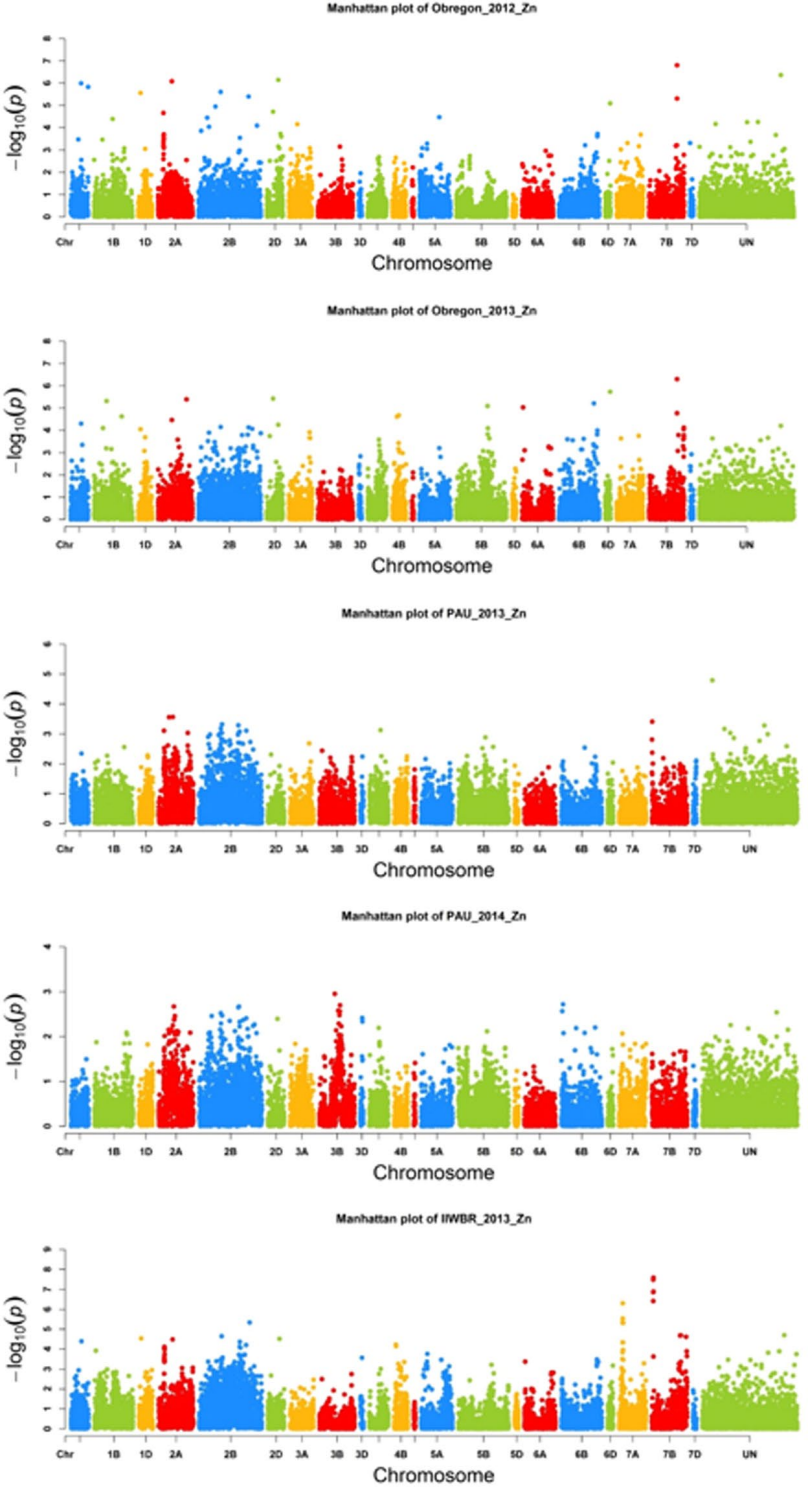
Significant SNPs (−10 log P values) associated with grain Zn from the Manhattan plot analysis of five promising environments.

**Figure 4:**
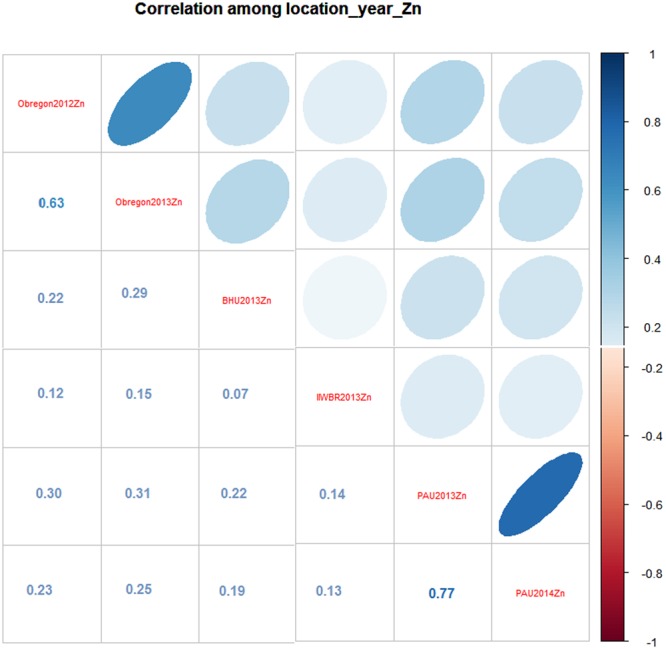
Correlation co-efficient between environments for grain Zn concentration.

A mixed linear model combining population structure and kinship data was used to identify a total of 39 significant MTA for grain Zn. Corresponding SNPs were assigned to 10 different locations on chromosomes 1A, 2A, 2B, 2D, 5A, 6B, 6D, 7B, 7D, and one uncertain chromosome (Table 1; Fig. 3). The SNPs explained 5–10.5% of phenotypic variation. Among the 39 MTA, seven were identified in at least three environments. A major SNP on chromosome 2A (*RAC875_c34757_180*) at 60 cM explained approximately 9% of the phenotypic variation while another SNP on chromosome 5A (*IAAV1375*) explained approximately 6%. For SNP *RAC875_c34757_180*, 277 lines had the CC genotype and 44 lines had the TT genotype. TT genotypes showed increased grain Zn concentration and thousand kernel weight (TKW). For SNP *IAAV1375*, 236 lines had genotype CC and 86 lines had genotype TT. The CC genotype displayed increased grain Zn and slightly reduced TKW (Fig. 4). Another major SNP, *wsnp_Ex_c5268_9320618*, was detected at 120 cM on chromosome 7B and explained approximately 10.5% of the variation.

**Table 1:** Significant stable SNPs identified for grain Zn across at least three different environments.

S. No.	Marker	Chromosome	Position	A Allele	B Alllele	p	F	additive_effect	additive_p	additive_F	dominance_effect	dominance_F	dominance_p	Marker R^2^	Genetic Variance	Residual Variance
1	Kukri_c12683_1309	1A	40	A	G	0.000	14.432	0.421	0.310	1.033	22.1	27.5	0.0	9.0	11.9	7.3
2	Kukri_c13363_675	1D	60	T	G	0.000	8.726	−0.903	0.172	1.872	7.8	15.4	0.0	5.5	10.2	16.3
3	BobWhite_c48481_81	2A	21	T	C	0.000	11.128	1.832	0.000	18.333	4.1	3.6	0.1	7.3	11.9	7.3
4	BS00022982_51	2A	21	T	C	0.000	8.448	−0.951	0.000	13.199	2.0	3.4	0.1	5.3	3.4	7.7
5	BS00070798_51	2A	21	T	C	0.000	8.077	−0.984	0.000	14.008	1.1	1.9	0.2	5.2	3.4	7.7
6	Excalibur_c92241_336	2A	21	T	G	0.000	9.752	0.971	0.000	13.496	2.4	5.8	0.0	6.3	3.4	7.7
7	GENE-0749_215	2A	21	A	G	0.000	8.159	1.637	0.000	14.794	1.5	1.8	0.2	5.2	11.9	7.3
8	Kukri_rep_c104307_905	2A	21	A	G	0.000	9.678	0.983	0.000	13.946	2.3	5.1	0.0	6.1	3.4	7.7
9	RAC875_c31045_882	2A	21	A	G	0.000	8.108	−0.934	0.001	12.332	2.0	3.4	0.1	5.1	3.4	7.7
10	RAC875_c34757_180	2A	60	T	C	0.000	14.643	−0.795	0.305	1.056	22.0	26.9	0.0	9.1	11.9	7.3
11	RAC875_rep_c78518_198	2A	21	A	G	0.000	8.260	1.687	0.000	15.563	1.6	1.5	0.2	5.3	11.9	7.3
12	wsnp_Ex_c15740_24096817	2A	21	A	G	0.000	9.435	0.951	0.000	13.194	2.5	5.3	0.0	5.9	3.4	7.7
13	BS00011149_51	2B	26	T	C	0.000	9.138	−0.356	0.401	0.708	5.7	18.0	0.0	5.9	11.9	7.3
14	Excalibur_c11392_1193	2B	76	A	G	0.000	8.800	0.858	0.004	8.367	3.2	12.9	0.0	5.6	3.4	7.7
15	Excalibur_c19649_1500	2B	78	A	G	0.000	10.379	−0.984	0.002	9.801	8.1	13.4	0.0	6.5	3.4	7.7
16	Excalibur_c23723_141	2B	60	T	C	0.000	10.590	−0.224	0.669	0.184	9.4	21.0	0.0	6.7	11.9	7.3
17	Excalibur_c26527_82	2B	135	T	G	0.000	9.719	−0.569	0.335	0.932	10.7	19.3	0.0	6.1	11.9	7.3
18	Excalibur_rep_c67411_210	2B	80	T	C	0.000	8.967	−0.718	0.270	1.223	14.5	17.8	0.0	5.7	10.2	16.3
19	Kukri_rep_c104810_341	2B	64	T	C	0.000	11.821	−0.969	0.076	3.180	11.9	19.4	0.0	7.4	11.9	7.3
20	RAC875_c18463_156	2B	66	A	G	0.000	13.468	−0.171	0.754	0.098	22.0	26.9	0.0	8.6	11.9	7.3
21	RAC875_c29913_139	2B	88	T	C	0.000	12.954	−0.333	0.393	0.731	22.0	25.1	0.0	8.3	11.9	7.3
22	wsnp_Ex_c20529_29609310	2B	70	A	C	0.001	7.525	0.731	0.297	1.090	8.9	14.6	0.0	4.8	10.2	16.3
23	wsnp_Ex_c9729_16071358	2B	76	A	G	0.000	9.747	0.865	0.004	8.515	3.7	14.6	0.0	6.1	3.4	7.7
24	Ku_c19185_1569	2D	40	A	G	0.000	12.997	0.387	0.651	0.205	15.3	26.0	0.0	8.1	10.2	16.3
25	Kukri_c14902_1112	2D	67	A	G	0.000	14.802	0.428	0.387	0.749	22.4	28.0	0.0	9.3	11.9	7.3
26	IAAV1375	5A	21	A	G	0.000	10.636	0.379	0.430	0.625	12.4	21.0	0.0	6.6	11.9	7.3
27	wsnp_Ex_c16551_25060833	5A	10	T	C	0.001	7.093	−0.904	0.001	10.599	2.7	3.5	0.1	4.5	3.4	7.7
28	Ra_c7673_1788	6B	100	A	C	0.000	9.472	0.503	0.435	0.612	12.5	18.8	0.0	6.0	10.2	16.3
29	wsnp_Ex_c7713_13153321	6B	75	A	C	0.001	7.784	0.250	0.594	0.284	9.5	15.1	0.0	4.8	10.2	16.3
30	Excalibur_c7546_1286	6D	122	A	C	0.000	13.759	0.284	0.575	0.315	18.1	27.3	0.0	8.7	10.2	16.3
31	BS00004376_51	7B	130	T	G	0.000	11.176	0.271	0.389	0.745	4.7	21.2	0.0	7.0	3.4	7.7
32	Excalibur_rep_c77206_397	7B	120	T	C	0.001	7.496	−0.337	0.489	0.480	8.9	14.2	0.0	4.7	11.9	7.3
33	RAC875_c34939_963	7B	140	T	G	0.000	11.007	0.064	0.813	0.056	5.1	21.9	0.0	6.9	3.4	7.7
34	RAC875_c525_1372	7B	142	T	C	0.000	8.943	0.356	0.502	0.452	9.4	17.6	0.0	5.6	10.2	16.3
35	Tdurum_contig44948_1132	7B	120	A	G	0.001	7.583	−0.352	0.468	0.528	12.0	14.3	0.0	4.7	11.9	7.3
36	wsnp_Ex_c5268_9320618	7B	120	T	C	0.000	16.469	−1.249	0.055	3.719	23.3	30.3	0.0	10.4	11.9	7.3
37	wsnp_Ex_c8400_14157318	7B	120	T	C	0.000	15.195	−1.205	0.130	2.304	18.7	29.0	0.0	9.5	10.2	16.3
38	RAC875_c104604_381	UN	1	T	C	0.000	10.083	0.048	0.938	0.006	12.1	19.9	0.0	6.3	11.9	7.3
39	wsnp_Ex_c24135_33382700	UN	1	T	G	0.000	15.369	0.786	0.090	2.897	22.7	28.9	0.0	9.9	11.9	7.3

There was a significant positive correlation between environments for grain Zn, except in BHU-2013 and IIWBR-2013. This suggests that, although there was significant G x E interaction, the ranking order of lines for grain Zn was maintained across locations (Fig. 4). Significant positive correlation was observed between environments for TKW and days to maturity (not shown). There was a strong correlation for grain Zn across both seasons at CENEB, indicating moderately-high heritability for grain Zn, though location or ecological zone may play an important role in determining micronutrient content as well as yield and yield-related traits. Genomic predictions were evaluated for accuracy using cross-validations approaches. The genomic predictions for grain Zn showed good potential, with correlations ranging from 0.5–0.65 for grain protein content, in accordance with the findings of Velu *et al*.^27^.

We also investigated the relationship between grain Zn and agronomic traits. The 347 markers (not shown) associated with days to heading, days to maturity, plant height, or TKW were not highly significantly associated with grain Zn. However, marker *Excalibur_c*2*3723_141* on chromosome 2B was associated with both TKW and grain Zn concentration. Based on these results, we conclude that high grain Zn is not related to agronomic traits or their pleiotropic effects (Table 2). These results suggest an absence of any confounding effects of *Ppd*, *Vrn*, and *Rht* genes. However, previous studies have shown that the *Rht* genes that confer semi-dwarf characteristics in CIMMYT wheat germplasm showed a reduction in grain Zn and Fe^6^, whereas a promising marker on chromosome 2B associated with TKW increased grain Zn concentration^19^. Positive associations between TKW and grain Zn concentration have also been reported in CIMMYT germplasm^19,28^.

**Table 2:** List of candidate genes associated with the stable SNPs identified.

S. No.	Marker	Chromosome	Position	p	Marker_R^2^	Genetic Variance	Linked genes	Candidate genes (closest/nearby)	Species
1	Kukri_c12683_1309	1A	40	0	9	11.9	TRIAE_CS42_7AS_TGACv1_571804_AA1849960	BTB/POZ domain-containing protein At3g05675-like	*Aegilops taushii*
2	RAC875_c34757_180	2A	60	0	9.1	11.9	TRIAE_CS42_2AS_TGACv1_112656_AA0342940	Na+/H+ antiporter (NHX2)	*T. aestivum*
3	BobWhite_c48481_81	2A	21	0	7.3	11.9	TRIAE_CS42_2AS_TGACv1_113144_AA0352040	Vacuolar protein sorting-associated protein 54	*Aegilops taushii*
4	RAC875_c18463_156	2B	66	0	8.6	11.9			
5	RAC875_c29913_139	2B	88	0	8.3	11.9	TRIAE_CS42_2DL_TGACv1_160628_AA0552420	D-3-phosphoglycerate dehydrogenase 1, chloroplastic-like	*Aegilops taushii*
6	Kukri_rep_c104810_341	2B	64	0	7.4	11.9			
7	Excalibur_c23723_141	2B	60	0	6.7	11.9	TRIAE_CS42_2BS_TGACv1_147153_AA0478820		
8	Excalibur_c19649_1500	2B	78	0	6.5	3.4	TRIAE_CS42_2BL_TGACv1_130672_AA0415740	BEACH domain-containing protein C2	*Aegilops taushii*
9	Kukri_c14902_1112	2D	67	0	9.3	11.9			
10	Ku_c19185_1569	2D	40	0	8.1	10.2	TRIAE_CS42_2DL_TGACv1_159449_AA0538480		
11	IAAV1375	5A	21	0	6.6	11.9	TRIAE_CS42_5AL_TGACv1_377519_AA1247650	Probable UDP-arabinose 4-epimerase 1	*Aegilops taushii*
12	Excalibur_c7546_1286	6D	122	0	8.7	10.2	TRIAE_CS42_6DL_TGACv1_526968_AA1696350		
13	wsnp_Ex_c5268_9320618	7B	120	0	10.4	11.9	TRIAE_CS42_7BL_TGACv1_578120_AA1889690	Zinc finger CCCH domain-containing protein 13	Brachpodium
14	wsnp_Ex_c8400_14157318	7B	120	0	9.5	10.2	TRIAE_CS42_7BL_TGACv1_579256_AA1905850	NF-X1-type zinc finger protein NFXL1-like	*Aegilops taushii*
15	BS00004376_51	7B	130	0	7	3.4	TRIAE_CS42_7BL_TGACv1_577483_AA1876500	TaMYB59 MYB-related protein	*T. aestivum*
16	RAC875_c34939_963	7B	140	0	6.9	3.4	TRIAE_CS42_7BL_TGACv1_578066_AA1888730		
17	wsnp_Ex_c24135_33382700	UN	1	0	9.9	11.9	TRIAE_CS42_2BL_TGACv1_131885_AA0433240	Protein IQ-DOMAIN 32	Brachpodium

### Genes encoding observed MTA

Significant SNPs identified from the GWAS study were used to locate known candidate genes relevant to metal homeostasis and transporters using the recently annotated wheat reference sequence (RefSeq V1.0). Interestingly, the major loci identified on chromosome 2 defined by the markers *BS00070798_51* and *RAC875_rep_c78518_198* is located in proximity to a heavy metal domain (metal ion transporter) and Glutathione S-transferase gene (TraesCS2A01G072500). Another marker on chromosome 2B (*Excalibur_c11392_1193*) is associated with a metal ion binding protein (Mg and Mn), which is a paralogue of a rice (*Oryza sativa* subsp *japonica*) metal binding protein gene ‘Os04g0609600’. This marker was linked with a phosphatase enzyme (Table 2). However, this marker exhibited the presence of SNP [A/G] in non-coding 3′ UTR region and did not exhibit any alteration in corresponding protein when the marker-linked cDNA was computationally translated into a putative protein. Further BLAST-N analysis revealed the higher conservation and 100% identity of this protein with *Aegilops taushii*, 90% with *T. urartu*, 92% with *Oryza sativa*, and more than 80% identity with other dicot species. This analysis revealed higher conservation of this gene from monocots to dicots and this marker is linked with an essential gene phosphatase for catalyzing phosphate ion cleavage on target proteins. It is presumed that higher phosphorous concentration competes with Zn accumulation and this phosphatase enzyme catalyzes phosphate and helps to accumulate more Zn in grain. Moreover, this phosphatase enzyme is an Mg^2+^ or Mn^2+^-dependent protein, which is highly conserved even from prokaryotes to eukaryotes.

One of the markers on chromosome 7B (*wsnp_Ex_c5268_9320618*) aligned with the database sequence from plants.ensembl.org. This marker was identified as ‘Zn finger motif’ with synonymous mutation. We expect that this transcription factor would not affect its own function and thus should not affect activation of downstream genes. This kind of genes is highly conserved and may sustain the function of other genes. Since it is ‘Zn finger motif’, a certain level of Zn is necessary for gene function, or if additional Zn becomes available the function of this transcription factor will be activated^29^.

**Figure 5:**
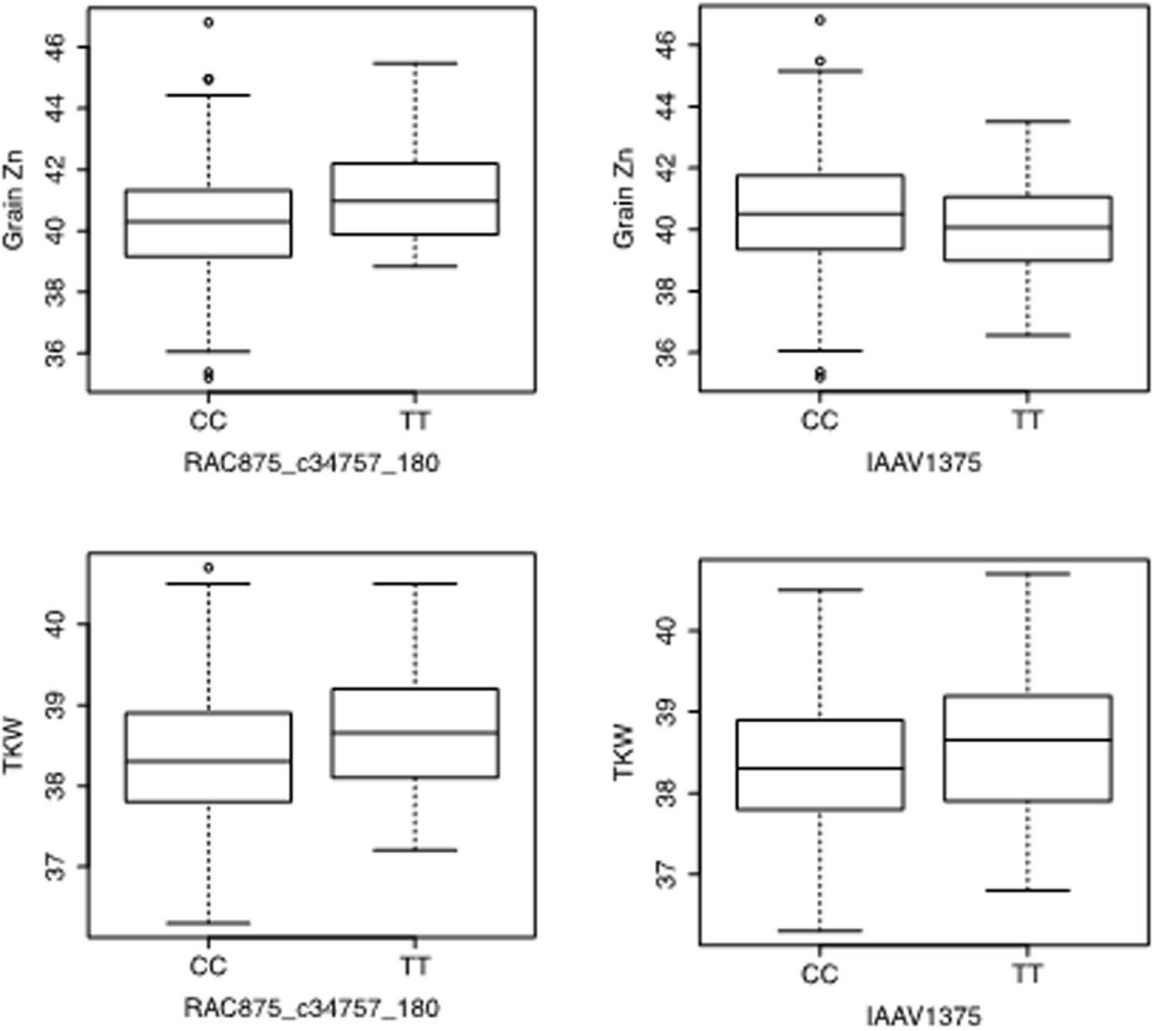
Boxplots of the relationship of grain Zn and TKW with polymorphism of markers *RAC875_c34757* and *IAAV1375*.

Interestingly, another marker on chromosome 6B (*Ra_c7673_1788*) was associated with ABC transporter G family (28-like). The ABC transporter in wheat is associated with pathogenic defense (for example, Lr34) and is critical for adaptation to stress environments^30^. Subsequent analysis suggests that allelic variation in SNPs (CC vs. TT) had appreciable positive effects on grain Zn and TKW for the SNP *RAC875_c34757_180* on chromosome 2B Fig. 5. In contrast, SNP *IAAV1375* on chromosome 5A had a positive effect on grain Zn but negative effect on TKW. Nevertheless, there was a positive pleiotropic effect of grain Zn with TKW, which is evident from the positive significant correlation between grain Zn and TKW^19,28^.

## Discussion

The diverse panel of 330 wheat lines were phenotyped in a range of environments in India and in Mexico and genotyped using the high-density iselect 90 K SNP assay to disclose the genetic mechanism of Zn accumulation in wheat grain. GWAS identified 39 MTA for grain Zn concentration, and associated candidate genes were localized using the sequence annotation (RefSeq v1.0) for finding the positions of the markers and the annotations of the genes. GWAS study identified two major effect QTL on 2 and 7 chromosome groups. A number of QTL for high grain Fe and Zn have been mapped on 2 and 7 chromosomes of wheat^19–22^. These results confirm that the group 2 and 7 chromosomes holding genes for nutrient uptake, translocation and sequestration of mineral in wheat plant^21,23^.

This study identified some promising genetic loci associated with grain Zn in wheat. Novel candidate regions, together with genes previously shown to regulate metal homeostasis genes, are among the promising genes for further validation and testing in CIMMYT wheat germplasm. Major QTL regions were identified on chromosomes 2B and 7B, concurring with previous studies and highlighting the potential of this region for fine mapping and gene discovery research. Our results indicate that the Zinc finger motif transcription factor and phosphatase (metal ion binding) play major roles in loading additional Zn to the wheat grain. Functional analysis is now being conducted for the selected markers using metabolomics approaches to give further information about Zn accumulation regulators and associated metal transport pathways. The markers and allelic combinations identified here can be used for marker-assisted selection. Fixing and accumulating these common loci and introducing new rare alleles into high-yielding elite germplasm represents a promising opportunity in breeding Zn biofortified wheat. In addition, genomic prediction based on all SNPs seems to be a more effective strategy than selection based on only a few SNPs. Genomic prediction approaches using dense DNA markers can therefore enable more efficient selection of lines at early stages of wheat breeding programs to reduce the breeding cycle compared to phenotyping.

Our study provides comprehensive use of wild progenitor species such as *T. spelta*, *T. dicoccum*, and *Ae. tauschii* for offering new source of alleles for high grain Zn in wheat. Sequences of significant SNP markers were aligned to contigs on publicly available databases (e.g. https://urgi.versailles.inra.fr/) to identify candidate genes from wheat, barley, Brachypodium, and rice. Furthermore, SNPs and corresponding gene sequences allowed us to anchor the some potential genomic regions (e.g. Zinc finger motif and metal ion binding genes) to recently-released wheat assemblies that are being investigated for genes involved in the biosynthesis and metal transport pathways. These genes, together with other known candidate genes, are being investigated and significant SNPs will be converted into Kompetitive Allele Specific PCR (KASP) markers that can be efficiently employed to transfer rare alleles into elite wheat lines. This study has demonstrated that modern genotyping technologies efficiently detect promising loci for nutritional quality traits. The development of PCR-based markers (such as KASP assays) can accelerate breeding through efficient marker-assisted selection and by pyramiding diverse QTL regions from different genetic resources.

Transcription factor candidate genes known to be associated with Zn accumulation which were mapped on chromosome 7B and probably associated with loci identified in bi-parental QTL analyses^21^. In addition, 18 SNPs identified in this study were co-located in/near regions of QTL for seed size. The co-location of QTL for Zn with seed size indicates that breeding for both higher micronutrients and seed size is highly possible. Marker-assisted selection for high Zn and Fe could simultaneously improve multi-associated target traits of seed size by using the co-associated genetic loci.

The rich genetic diversity for Zn in various wheat wild species and landraces provides novel alleles for genetically enhancing wheat Zn and Fe. Traditional breeding approaches have been used to successfully incorporate several novel alleles for grain Zn into elite germplasm by crossing high yielding elite wheat lines with *Ae. tauschii*-based synthetic hexaploid wheats or *T. spelta* accessions. CIMMYT’s biofortification breeding program has incorporated several novel alleles for grain Zn in elite, high-yielding germplasm via targeted crosses with increased population sizes and by selecting for agronomic traits in early generations^14^. The result has been the development and release of biofortified wheat varieties such as ‘Zinc Shakti (Chitra)’, WB-02, HPBW 01 (PBW 1 Zn), Zincol-2016, and BARI-Gom 33, which possess approximately 30–40% higher grain Zn than local checks^15^.

While grain Zn concentration is under quantitative genetic control and influenced by soil and other associated factors, it can be further improved by pyramiding multiple grain Zn QTL in high yielding wheat. Although grain Zn expression influenced soil and environmental factors, moderately high heritability and high genetic correlations between environments suggests ranking of high Zn genotypes across diverse environments^14^. The genetics of biofortification traits is now better understood and promising genotypes that combines high Zn density and stable performers are being used in the biofortification breeding program.

High-throughput, non-destructive phenotyping for grain Zn and Fe using EDXRF analysis is facilitating selection^31^. Gene discovery and mapping studies leading to the utilization of markers to further improve the breeding efficiency. As proof of concept of germplasm deployment of improved germplasm simultaneously enriching genetic knowledge through high-density genomics analysis could enhance breeding efficiency.

## Methods

### Plant materials and phenotypic data collection

The HPAM panel consists of 330 wheat lines from CIMMYT’s biofortification breeding program, as described previously by Velu *et al*.^27^. The HPAM panel was evaluated for grain Zn concentrations and other agronomic traits at the Norman E. Borlaug Experiment Station (CENEB), Ciudad Obregon (27°24′N, 109°56′W), Mexico, during two successive crop seasons (2011–12 and 2012–13). Mega-variety PBW 343 and Waxwing (Attila*2/STAR, Attila is a sibling of PBW 343) were used as checks. The experiment was conducted during the 2012–13 and 2013–14 winter seasons (November to April) and utilized a randomized complete block design (plot size 2 m^2^) with two replications. The HPAM panel was also planted during the 2012–13 and 2013–14 winter seasons at Punjab Agricultural University (PAU), Ludhiana, India, and 2012–13 crop season at Banaras Hindu University, Varanasi, India (BHU), and at the Indian Institute of Wheat & Barley Research, Karnal, India (IIWBR), using the same layout as in Mexico. Soil Zn content heterogeneity is a historic constraint at CENEB, resulting in the application of 25 kg ha^−1^ of ZnSO_4_ every season since 2009–10 to enrich the available soil Zn and minimize Zn heterogeneity. Sufficient nutrition and water were supplied throughout the trials to avoid potential nutrient and drought stresses. Fungicide and pesticides were applied to protect the crop from pest and diseases. Field observations were recorded for TKW, plant height, days to heading and days to maturity.

### Grain sampling and trait determination

Plant materials were harvested after physiological maturity when grains were totally dry in the field. Grain samples (approx. 20 g/entry) were carefully cleaned, with broken grains and foreign materials discard, and the residual sample was used for micronutrient analysis. Grain samples from all five environments were analyzed using a ‘bench-top’, non-destructive, energy-dispersive X-ray fluorescence spectrometry (EDXRF) instrument (model X-Supreme 8000, Oxford Instruments plc, Abingdon, UK) that was standardized for high-throughput screening of grain Zn and Fe concentration (unit: mg/kg) in wholegrain wheat^31^. A proficiency study was conducted by Flinders University, Adelaide comparing EDXRF instrument results with the original Inductively Coupled Plasma (ICP) data which confirmed that the EDXRF results are reproducible. Soil analysis of the experimental area showed average Zn concentration of 1.3 ppm at 0–30 cm depth and 0.78 ppm at a soil depth of 30–60 cm.

### Statistical models

Broad-sense heritability (*H*^*2*^) was estimated across environments, using the formula ${H}^{2}=\sigma {g}^{2}/(\sigma {g}^{2}+\sigma g{e}^{2}\mathrm{/1}+\sigma {e}^{2}/rl)$, where *σg*^2^ is the genotypic variance, *σge*^2^ is the genotype × environment variance, and *σe*^2^ is the residual error variance for r replicates and l locations using the R program. Genotypic values (i.e. line means) were estimated as Best Linear Unbiased Estimators as fixed effect, with a random effect for replicates nested within each environment.

### Genotypic data and GWAS

DNA was extracted according to standard CTAB procedures and genotyped using the Illumina iSelect 90 K Infinitum SNP array developed in wheat^25^. The array revealed a total of 28,074 SNP markers, which we coded as 0 and 1 for homozygote and 0.5 for heterozygote allele scores for data analysis. Markers were filtered for 70% missing data, 0.10 minor allele frequency, and greater than 10% heterozygosity, giving a remaining total of 14,273 SNP markers for GWAS.

Significant MTAs were identified using the mixed linear model approach in TASSEL5.0, which simultaneously accounted for population structure and kinship. Population structure (summarized by the Q matrix) was inferred by the STRUCTURE program v.2.2, and the kinship matrix (summarized by the K matrix) was calculated using TASSEL5.0. SNPs with *p* ≤ 0.0001 were considered significantly associated with individual traits. *R*^2^ was used to evaluate the magnitude of MTA effects. The recent version of IWGSC RefSeq v1.0 have been accessed for identifying associated candidate genes.
